# Low Tongue Strength and the Number of Teeth Present Are Associated with Cognitive Decline in Older Japanese Dental Outpatients: A Cross-Sectional Study

**DOI:** 10.3390/ijerph17228700

**Published:** 2020-11-23

**Authors:** Rui Egashira, Shinsuke Mizutani, Masahiro Yamaguchi, Tomotaka Kato, Yojiro Umezaki, Saori Oku, Keiko Tamai, Toyoshi Obata, Toru Naito

**Affiliations:** 1 Section of Geriatric Dentistry, Department of General Dentistry, Fukuoka Dental College, Fukuoka 814-0193, Japan; egashira@college.fdcnet.ac.jp (R.E.); yamagum@college.fdcnet.ac.jp (M.Y.); umezaki@college.fdcnet.ac.jp (Y.U.); tamai@college.fdcnet.ac.jp (K.T.); naito@college.fdcnet.ac.jp (T.N.); 2 OBT Research Center, Faculty of Dental Science, Kyushu University, Fukuoka 812-8582, Japan; 3 Section of Geriatric Dentistry and Perioperative Medicine in Dentistry, Division of Maxillofacial Diagnostic and Surgical Sciences, Faculty of Dental Science, Kyushu University, Fukuoka 812-8582, Japan; 3dd16002p@s.kyushu-u.ac.jp; 4 Department of Oral Health Sciences, University of Washington, Seattle, WA 98195, USA; tomo-k@tky.ndu.ac.jp; 5 Obata Medical Clinic, Fukuoka 814-0175 Japan; zyuzenn@msn.com

**Keywords:** cross-sectional study, elderly, oral function, cognitive function, geriatric dentistry

## Abstract

To mitigate the impact of dementia, initiating early intervention is important. This study aims to investigate the associations between deterioration in oral function and cognitive decline in older outpatients whose oral health was maintained in the dental clinic. This study included 50 outpatients aged ≥65 years. We used the Japanese version of the Montreal Cognitive Assessment (MoCA-J) to assess cognitive decline. Oral function was evaluated by tongue pressure, masticatory performance, and swallowing ability. A full-mouth periodontal examination was conducted, and the occlusal support and number of teeth were recorded. Odds ratios (ORs) and 95% confidence intervals (CIs) for cognitive decline (MoCA-J score ≤25 points) were calculated using logistic regression models. The age, number of teeth, tongue pressure, and masticatory performance were significantly correlated with cognitive decline (*p* < 0.05). Logistic regression analyses revealed that cognitive decline was independently associated with age (OR: 1.25; 95% CI: 1.03–1.52; *p* = 0.024), number of teeth (OR = 0.83; 95% CI: 0.76–1.00; *p* = 0.047), and lower tongue pressure (OR: 0.87; 95% CI: 0.77–0.98; *p* = 0.022). Lower tongue pressure and a small number of remaining teeth may be associated with cognitive decline in Japanese outpatients.

## 1. Introduction

Currently, the number of patients with dementia worldwide is approximately 50 million; projections suggest that this population will be 82 million in 2030 and 152 million in 2050 [1]. While many new treatments are under investigation in various clinical trial stages, currently no treatment has been approved for use that is effective in curing dementia or limiting its progressive course [2]. Thus, it is warranted to explore means to prevent the onset of dementia. Various risk factors such as smoking, social isolation, depression, and low educational levels have been revealed, and several preventive measures have been identified [3,4,5]. These studies have also mentioned that the early detection and treatment of mild cognitive impairment (MCI) is key in preventing dementia.

MCI is the intermediate stage between normal cognitive aging and dementia, and the remarkable point is its reversibility [4]. The analysis of reversion rates from 25 studies included in a meta-analysis suggested an overall reversion rate of approximately 24% [6]. However, it is unclear which factors are related to successfully returning to normal cognitive aging.

Previous epidemiological studies have explored the relationship between dementia and oral health. Lee et al. [7] reported that periodontitis is associated with a high risk of developing dementia, whereas Tamaki et al. [8] indicated that oral hygiene is associated with cognitive function. Furthermore, certain studies showed that tooth loss is associated with the onset of dementia, particularly in the Japanese population [9,10].

Moreover, recent studies have suggested relationships between MCI and periodontitis, gingival inflammation, oral motor skills, occlusal force, and occlusion contacts [11,12,13,14]. Of note; however, a previous epidemiological survey revealed that most studies performed a simple evaluation of oral status or periodontal disease; for example, the data on the number of teeth and masticatory functioning were reviewed using a self-administered questionnaire [10]. Moreover, the oral assessment used in each study was not comprehensive but rather restrictive. In other words, few studies seem to have evaluated oral status and function, such as tongue pressure, masticatory performance, and swallowing, yet these studies revealed a relationship between MCI and various measures of oral status and function. Moreover, these epidemiological studies targeted community-dwelling older populations, with few available reports that assessed dental outpatients whose oral health was maintained by dentists and dental hygienists.

Thus, we hypothesized that not only conventional oral diseases such as dental caries or periodontitis but also oral function may be associated with cognitive decline. The present study sought to examine whether deterioration in oral function is associated with a decline in cognitive function in older outpatients whose oral health concerns, such as dental caries or periodontitis, are treated.

## 2. Materials and Methods

### 2.1. Participants

**Figure 1:**
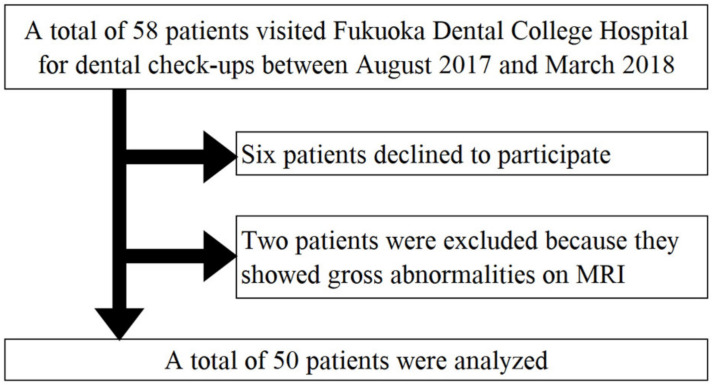
Flowchart of the study participants.

Fifty-eight outpatients, who regularly received dental care from dentists and dental hygienists, were recruited in a consecutive sample between August 2017 and March 2018 at the General Dentistry and Geriatric Dentistry Department of Fukuoka Dental College Hospital. They were males and females aged ≥65 years who had not been previously diagnosed with dementia. Some outpatients with chronic conditions, such as hypertension, were included; however, those with acute oral symptoms or a serious illness, such as cancer, were excluded. Six patients declined to participate in our study. Furthermore, two of the 52 participants were later excluded because they showed gross abnormalities on brain magnetic resonance imaging. Therefore, data from 50 participants (mean age ± SD, 71.5 ± 3.8 years) were finally analyzed (Figure 1). Kyushu University Institutional Review Board for Clinical Research (Approval number: 30–105) and the Ethics Committee of Fukuoka Gakuen (Approval number: 405) approved the study. The investigations were performed following the guidelines of the Declaration of Helsinki of 1975. Trial registration number: UMIN000033380.

Name of registry: Study of the relationship between oral function and cognitive decline in elderly people

URL of registry: https://upload.umin.ac.jp/cgi-open-bin/ctr/ctr_view.cgi?recptno=R000038059

Date of registration: 14 January 2020

Date of enrolment of the first participant to the trial: 1 August 2017

### 2.2. Cognitive Function

The Japanese version of the Montreal Cognitive Assessment (MoCA-J), which is a simple cognitive screening tool available for detecting MCI in older people, was used to assess cognitive function. The MoCA-J is a one-page test that takes approximately 10 min to administer, the total score is in the range of 0–30 points, and a score of ≥26 points suggests that the respondent is healthy. If the participant has ≤12 years of education, one point is added to his/her total score. The sensitivity and specificity of MoCA-J results for detecting MCI in Japanese people were reported as 90% and 87%, respectively [15]. The primary study outcome was cognitive decline according to the MoCA-J score.

### 2.3. Oral Examination

An oral examination was performed by one of four trained dentists. In each participant, after counting the number of teeth in the mouth, probing pocket depth, clinical attachment level, and bleeding on probing were assessed at six sites (the mesiobuccal, mid-buccal, distobuccal, mesiolingual, mid-lingual, and distolingual surfaces of each tooth). Using these measurements, the periodontal inflamed surface area (PISA) [16], which reflects the amount of inflamed periodontal tissue, was calculated using a dedicated calculation sheet. The participants were also classified according to the Center for Disease Control and Prevention (CDC)/American Academy of Periodontology case definitions proposed by the CDC Working Group [17]. Furthermore, the existence of remaining natural occlusal supports was examined using the Eichner classification, and the study participants were subsequently divided into three groups as follows: group A, intermaxillary contact in four occlusal supporting zones (i.e., in the premolar and molar regions); group B, intermaxillary contact not in all occlusal supporting zones; and group C, no intermaxillary contact.

### 2.4. Oral Function

Maximal tongue pressure was evaluated using a tongue pressure measurement device (JMS tongue pressure device; JMS Co., Ltd., Hiroshima, Japan) [18]. A small balloon that was attached to the tip of a probe was placed in the participant’s mouth and the participant was instructed to close their lips, raise their tongue, and compress the balloon onto the hard palate with maximal spontaneous effort for approximately 5 s. Tongue pressure values were recorded three times and the maximal value was used for analysis [19].

Masticatory performance was assessed using masticatory performance gum (Masticatory Performance Evaluating Gum XYLITOL; Lotte Co., Ltd., Tokyo, Japan). The gum changes its color from green to red when chewed. Participants were asked to chew the gum for 1 min without restrictions on the side of the mouth they used to chew [20]. The chewing gum was wrapped with polyethylene film and flattened to a thickness of 1.5 mm between two glass plates, and we measured L*, a*, and b* using a colorimeter (CR-20; Konica-Minolta Sensing, Tokyo, Japan). L* represented brightness of color, a* represented the degree of color between red and green, and b* represented the degree of color between yellow and blue. Changes in color were visualized as three-dimensional coordinates organized along L*, a*, and b* axes and then assessed using the International Commission on Illumination (CIE) LAB color system [21]. Differences between two colors (before and after gum chewing) in the CIELAB color space (ΔE) were calculated using the following equation. We measured L*, a*, and b* values of the gum before chewing, and the mean values were 72.3, −14.9, and 33.0, respectively.

$$\Delta E=\sqrt{{\left(L*-72.3\right)}^{2}+{\left(a*+14.9\right)}^{2}+{\left(b*-33.0\right)}^{2}}$$

The repetitive saliva swallowing test (RSST) was separately used to assess swallowing ability. Participants were asked to sit in a relaxed position and swallow as many times as possible for 30 s. An RSST count of ≤2 swallows in 30 s indicated suspected dysphagia [22]. The sensitivity and specificity of RSST in forecasting aspiration during video fluorographic examination were 0.98 and 0.66, respectively [23].

### 2.5. Data Collection for Other Variables

Participants answered a questionnaire provided by one of four trained dentists. Aside from sex and age, the questionnaire also included questions on medical history, medication, dental habits (frequency of brushing teeth, use of dental floss or picks, and regular dental check-ups), and smoking status (current/never/former) [24]. Moreover, participants’ body mass index (BMI) values were calculated using their height and weight measurements. The Geriatric Depression Scale (GDS) has been tested and used extensively in the older population [25]. In this study, a short form of the GDS (GDS-SF) was administered, consisting of 15 yes–no questions that could be completed quickly [26]. Here, a score of zero to four points was considered normal; five to eight points suggests mild depression; nine to 11 points suggests moderate depression; and 12 to 15 points was almost always indicative of depression.

### 2.6. Sample Size Calculation

To our knowledge, the association of the MoCA-J score with tongue pressure in dental outpatients has not been reported yet. However, the correlation between the mini-mental state examination score and tongue pressure has been analyzed (r = 0.33) [27]. The sample size was estimated using G* Power (ver. 3.1.9.4, Universität Kiel, Kiel, Germany), and minimum sample sizes were calculated using a correlation test because both the MoCA-J score and tongue pressure are continuous variables. Considering an effect size of 0.35, α of 0.10, and power (1 − β) of 0.80, a minimum sample size of 46 was required.

### 2.7. Statistical Analysis

First, participants were divided into two groups according to their MoCA-J score as being healthy (MoCA-J score ≥26 points) or showing cognitive decline (MoCA-J score ≤25 points). Second, the Mann–Whitney U test or chi-squared test including Fisher’s exact test as an option was used to determine any significant differences between the healthy and cognitive decline groups. Using a logistic regression model, both odds ratios (ORs) and 95% confidence intervals (CIs) were calculated. The groups (healthy vs. cognitive decline) were used as dependent variables. Independent variables were selected when the *p*-value was <0.05 in the Mann–Whitney *U* test or chi-squared test for each variable. Age (continuous), number of teeth (continuous), tongue pressure (continuous), and masticatory performance (continuous) were added as independent variables to the multivariate analysis (backward stepwise selection method). However, masticatory performance was excluded from the final model because no significant differences were found. We used the Hosmer–Lemeshow test for evaluating the goodness of fit of logistic regression models. *p* < 0.05 was considered to be statistically significant. Analyses were performed using the Statistical Package for the Social Sciences version 26.0 software program (IBM Corp., Armonk, NY, USA).

## 3. Results

Of 50 participants, 19 participants (38%) showed cognitive decline (MoCA-J score ≤25 points). Table 1 shows a comparison of the variables in the two groups stratified by MoCA-J score. The median age (25th percentile–75th percentile) was 70 (68–73) years in the healthy group and 74 (70–76) years in the cognitive decline group (*p* = 0.008). Participants in the cognitive decline group were significantly older than those in the healthy group. The most common conditions reported in participants’ medical histories were hypertension (34%), dyslipidemia (16%), and diabetes mellitus (16%). There were no significant differences between the two groups in the number of medicines and dental habits.

Table 2 compares the oral status and function between the two groups. Overall, no participants in this study had “severe” periodontitis. The two groups did not significantly differ in oral status except for in the number of remaining teeth. However, masticatory performance and tongue pressure were significantly lower in the cognitive decline group than in the healthy group (*p* = 0.035 and *p* = 0.023, respectively).

Following adjustment for confounding factors in the logistic regression model, cognitive decline was independently associated with older age (OR: 1.25; 95% CI: 1.03–1.52; *p* = 0.024), number of teeth (OR: 0.83; 95% CI: 0.76–1.00; *p* = 0.047), and lower tongue pressure (OR: 0.87; 95% CI: 0.77–0.98; *p* = 0.022) (Table 3). The accuracy of discrimination was 62.0%, whereas the Hosmer–Lemeshow test found the model fit to be acceptable, with a chi-squared statistic of 9.180 (*p* = 0.327).

## 4. Discussion

In this study of older Japanese outpatients, the nature of associations between cognitive decline and oral factors were investigated. Although previous studies have described the relationships between cognitive decline and occlusal force, periodontitis, periodontal inflammation, and lip movement among community-dwelling individuals [12,13,14], to our knowledge, this is the first study to investigate the association between low tongue pressure, number of remaining teeth, and cognitive decline in patients who visited the dental clinic to maintain oral health.

Two recent systematic reviews concluded that existing evidence pertaining to the association between oral health and cognitive status is weak. However, these systematic reviews primarily focused on oral hygiene and cognitive function in elderly individuals, with little focus on oral function. Therefore, more evidence is needed in this regard [28,29]. Moreover, other systematic reviews have concluded that the evidence available to support strategies for improving dental health in older patients with cognitive impairment was insufficient [30,31]. Thus, further evidence regarding the relationship between oral health—especially oral function—and cognitive decline is needed; consistent with this, the present results could provide new insights into the nature of correlations between oral functions and cognitive decline. Furthermore, since this study targeted outpatients whose oral health was regularly maintained in the dental clinic, all participants were expected to have similar factors that could influence the degree of cognitive decline, such as education, income, and activities of daily living. Therefore, our study may suggest that this population is suitable for investigating the association between oral function and cognitive functions while excluding other related factors that influence the onset of cognitive decline.

The mechanism underlying the relationship between cognitive decline and decreased tongue pressure currently remains unclear. An epidemiological study has shown that decreased lip movement was related to MCI. They have also reported that decreased lip movement caused unclear speech, which may cause more difficulty in conversation [13]. Another study on community-dwelling individuals found that those with higher tongue pressures were more socially active than those with lower tongue pressures [19]. On the other hand, social activities for the elderly reduced their risk of cognitive decline [32]. Thus, the deterioration of oral function might be associated with cognitive decline resulting from decreased rates of activity. A study revealed that social activity was related to the participation of preventive health services [33]. In this study, the influence of social activity or isolation was not examined, but our participants were considered more active than general community-dwelling individuals because they experienced regular dental check-ups. Thus, both tongue pressure and cognitive function in our participants may be higher than those in community-dwelling individuals. Further studies must be designed to evaluate the effect of oral function on activity levels.

In recent times, considerable attention is focused toward the assessment of tongue pressure in the fields of gerontology and gerodontology. For instance, a study indicated that tongue pressure was associated with a higher risk of general frailty [34] or sarcopenia [35]. The tongue performs complicated movements to facilitate mastication, swallowing, and speech in conjunction with the muscles in the oral cavity, mandible, larynx, and pharynx [36,37]. Decreased tongue pressure may affect normal mastication, bolus formation, and swallowing, which (considering their negative impact on the quantities or volumes of food/liquids consumed) may, in turn, be responsible for malnutrition [35,38,39]. A study indicated that maximal occlusal force was positively associated with cognitive function directly as well as indirectly via dietary intake [12]. Because tongue pressure as well as occlusal force crucially contribute to nutrient intake, the assessment of tongue pressure may be clinically important in reducing the risk of a decline in cognitive function, which may contribute to preventing the onset of MCI.

The previous review concluded that almost all studies reported the association between mastication and cognitive state [40]. In our study, the number of teeth was associated with cognitive decline but masticatory performance was not. This discrepancy might be because of differences in the assessment of masticatory performance, cognitive function, and the target population. Since our participants were dental outpatients who received regular check-ups, they did not have mastication disorders. However, a study has shown that tooth loss reduces the input of periodontal mechanoreceptors from the trigeminal nerve, which affects the hippocampus-dependent cognitive function [41]. Another study has focused on the effect of the impaired neurotransmission on changes in the trigeminal nervous system-related neural pathways to the hippocampus [42]. Therefore, it may be important to maintain oral health so that the brain can be stimulated through the periodontal membrane.

In this study, participants in the cognitive decline group were significantly older than those in the healthy group (Table 1) and increased age was a risk factor for cognitive decline (Table 3). Given that many studies have suggested the relationship between aging and cognitive decline, this finding seems reasonable [43]. Our study did not show an association between BMI and cognitive decline; however, a previous study has revealed this association [44]. This difference may be attributable to the study population. The previous study was performed in Israel and the average BMI of participants was 25.6 ± 3.5; in our study, there were no obese participants (obesity: BMI ≥ 30) and the average BMI of our participants was 22.4 ± 2.7.

Previous epidemiological studies have shown associations between MCI and periodontitis, gingival inflammation, and occlusion contacts or functional masticatory units [11,14,45], but the present results did not support such associations. This discrepancy may be attributable to the differences in the study population because our participants were outpatients and their oral health was being managed by dentists and dental hygienists. A preceding review indicated three possible mechanisms for the relationship between periodontitis and cognitive impairment: (1) A direct process via the bloodstream; (2) an indirect process via inflammatory mediators; and (3) the induction of the expression of platelet aggregation proteins [46]. One of two recent reviews concluded that chronic periodontitis resulting in tooth loss, but not before the inflammation, has affected the central nervous system by impairing cognition [47]. The other review indicated that the causal relationship between periodontitis and Alzheimer´s disease is unknown [48]. Because our study was cross-sectional, the causal relationships between tooth loss (loss of occlusion contacts), periodontitis, and cognitive impairment were unclear. Therefore, although there was no direct link between occlusal supports and cognitive decline in this study, the current results should be interpreted with some caution. Further cohort investigation may be needed to elucidate serological markers such as tumor necrosis factor-α and interleukin-6.

Epidemiological studies have also suggested the existence of relationships between cognitive decline and hypertension, dyslipidemia, and diabetes [49,50]. In contrast, there were no such associations found in our study. However, patients who receive regular dental check-ups tend to have a high level of health awareness [51]. In fact, no participants in our study were current smokers or obese. Therefore, it is possible that the participants of this study were more aware of their health, indicating an association between cognitive decline and lifestyle-related diseases such as hypertension. Thus, we have to consider the effect of selection bias in our study.

MoCA-J is a useful means to measure comparative cognitive functional decline, including that associated with physiological aging. Although we adopted a guideline of ≥26 points to indicate a healthy individual, which is generally recognized in Japan, the cut-off point of MoCA-J score may be affected by culture. A previous study suggested that ethnicities and educational systems are heterogeneous in developing countries, which can cause cultural differences [52]. The Japanese socioeconomic status is relatively homogeneous, so there is little variability in determining MCI using MoCA-J. Moreover, the level of participants’ cognitive decline was not diagnosed, although the MoCA-J is suitable and very useful as a convenient screening tool. Many studies have used MoCA-J as the primary outcome [12,53], but our study is limited in that MCI was not diagnosed by neurologists.

In contrast, the advantages of this study include that the targets were outpatients and trained dentists evaluated their oral status and function (as compared with self-assessment). Furthermore, the assessment of oral function included various items such as masticatory performance and swallowing ability, which are essential for food intake and quality of life, especially in older adults [54]. In Japan, a country with one of the world’s most rapidly aging populations, oral hypofunction has begun to attract attention in not only the field of dentistry but also in the medical and caregiving fields [37]. Recovery from oral functional decline can be expected with various interventions. Moreover, tongue pressure might be improved with training [55]. Thus, improving tongue pressure by following dentist instructions and self-care efforts may facilitate the maintenance of cognitive function. Furthermore, tongue pressure might be a potential marker for the early detection of MCI in the dental clinic.

The present study has some limitations. First, because this study was cross-sectional rather than longitudinal in nature, whether low tongue pressure affected cognitive decline remains uncertain. Prospective cohort studies may provide information beyond what was recorded here. Second, all participants were outpatients who visited the Fukuoka Dental College Hospital in Japan for regular dental check-ups, which may limit the ability to extrapolate these findings to the general Japanese population of older people. Third, when we compared the tongue pressure between the healthy and cognitive decline groups, the statistical power was 0.77. The insufficient value may have resulted from the relatively small sample size. Moreover, the frequent outcome (cognitive decline) may have caused an overestimation of the OR. Therefore, future studies should enroll more participants for effective evaluation of various factors. For instance, the association between tongue pressure and dental arch form has been previously described [56]. Finally, the oral function assessed in this study required voluntary movements by the participants. Although these assessments were also used for patients with cognitive decline [57], varying levels of cognitive decline might affect the measurement results.

In recent years, several studies have suggested the existence of a relationship between oral function and cognitive decline. However, few have evaluated possible associations between the function of a specific oral organ and cognitive decline. This study indicated that a small number of teeth and low tongue pressure are associated with cognitive decline. These findings provide the base for further research including interventional trials to evaluate whether improved tongue strength may prevent cognitive decline.

## 5. Conclusions

Previous epidemiological studies have targeted community-dwelling older individuals [12,13,14]. This is the first investigation to examine dental outpatients who regularly visited a dental hospital and whose oral health was managed by dentists and dental hygienists. The results of this study indicate that lower tongue pressure and a small number of teeth may be associated with cognitive decline. Our results suggest that the maintenance of oral functions such as tongue strength is necessary in older patients who visit the dental clinic. Furthermore, tongue pressure and the number of teeth might be potential markers for the early detection of MCI in the dental clinic.

## Figures and Tables

**Table 1:** Comparison of variables in the two groups stratified by MoCA-J score.

Variables		Healthy (*n* = 31)	Cognitive Decline (*n* = 19)	*p*-Value
Age (y)		70 (68–73)	74 (70–76)	0.008 ^†^
Sex	Male	10 (32)	4 (21)	0.522 ^‡^
	Female	21 (68)	15 (79)	
Body mass index (kg/m^2^)		22.7 (21.1–24.1)	22.8 (18.8–24.7)	0.770 ^†^
Medical history				
Hypertension	Yes	11 (36)	6 (32)	1.000 ^‡^
	No	20 (65)	13 (68)	
Dyslipidemia	Yes	5 (16)	3 (16)	1.000 ^‡^
	No	26 (84)	16 (84)	
Diabetes	Yes	6 (19)	2 (11)	0.693 ^‡^
	No	25 (81)	17 (90)	
Number of medicines		1 (1–2)	1 (1–2)	0.915 ^†^
Education level (y)		12 (12–14)	12 (12–14)	0.242 ^†^
GDS-SF		2 (1–3)	3 (1–6)	0.206 ^†^
Tooth brushing (time/day)	1	11 (35)	7 (37)	0.731 ^‡^
	2	19 (61)	12 (63)	
	3	1 (3)	0 (0)	
Use of dental floss or picks	Yes	24 (77)	18 (95)	0.134 ^‡^
Regular dental check-ups	Yes	31 (100)	19 (100)	
Smoking habit	Current	0 (0)	0 (0)	0.757 ^‡^
	Former	10 (32)	5 (26)	
	Never	21 (68)	14 (74)	

Values are reported as numbers (percentages) or medians (25th–75th percentile). MoCA-J: The Japanese version of the Montreal Cognitive Assessment, GDS-SF: Geriatric Depression Scale short form. † Mann–Whitney U test, ‡ Fisher’s exact test or Chi-square test.

**Table 2:** Association between oral factors and cognitive decline in the two groups stratified by MoCA-J score.

Variables		Healthy (*n* = 31)	Cognitive Decline (*n* = 19)	*p*-Value
Number of teeth		25 (22–27)	21 (18–24)	0.014 ^†^
Occlusal supports	Group A	19 (61)	8 (42)	0.345 ^‡^
	Group B	11 (36)	10 (53)	
	Group C	1 (3.2)	1 (5.3)	
Periodontitis	Moderate	8 (26)	6 (32)	0.814 ^‡^
	Mild	14 (45)	7 (37)	
	No periodontitis	9 (29)	6 (32)	
PISA (mm^2^)		174 (147–302)	175 (130–231)	0.569 ^†^
Masticatory performance (ΔE)		33 (29–39)	30 (23–37)	0.035 ^†^
Swallowing	RSST < 3	1 (3.2)	3 (16)	0.147 ^‡^
	RSST ≥ 3	30 (97)	16 (84)	
Tongue pressure (kPa)		35 (31–39)	31 (24–36)	0.023 ^†^

Values are reported as numbers (percentages) or medians (25th–75th percentile). MoCA-J: The Japanese version of the Montreal Cognitive Assessment, PISA: Periodontal inflamed surface area, RSST: The repetitive saliva swallowing test. † Mann–Whitney U test, ‡ Fisher’s exact test.

**Table 3:** Adjusted odds ratios and 95% confidence intervals for cognitive decline (MoCA-J ≤ 25 points).

Variables	Odds Ratio	95% Confidence Interval	*p*-Value
Age	1.25	1.03–1.52	0.024
Number of teeth	0.83	0.76–1.00	0.047
Tongue pressure	0.87	0.77–0.98	0.022

Model fit (backward elimination method): Hosmer–Lemeshow test (*p* = 0.327) and the accuracy of discrimination was 62.0%. Masticatory performance was excluded from the final model because there was no significant difference. Dependent variable: cognitive decline (0: MoCA-J score >25, 1: MoCA-J score ≤25). Independent variables: Age (continuous), number of teeth (continuous), and tongue pressure (continuous). MoCA-J: The Japanese version of the Montreal Cognitive Assessment.
